# Clinical predictors of prognosis in patients with advanced non-small cell lung cancer receiving immunotherapy

**DOI:** 10.1097/MD.0000000000046949

**Published:** 2026-01-23

**Authors:** Zhian Qiao, Lin Pang, Hongxin Zheng

**Affiliations:** a Radiation Therapy Department, Xingtai People’s Hospital, Xingtai, Hebei Province, China.

**Keywords:** immune checkpoint inhibitors, immunotherapy, logistic regression, non-small cell lung cancer, prognostic factors

## Abstract

This study aimed to investigate clinical predictors of prognosis in patients with advanced non-small cell lung cancer (NSCLC) receiving immunotherapy. A total of 122 patients with stage IIIB to IV NSCLC who received immune checkpoint inhibitor-based therapy between August 2020 and August 2024 were retrospectively analyzed. Eligible patients were ≥18 years old and received at least 1 cycle of immune checkpoint inhibitor monotherapy or combination therapy. Baseline demographic, clinical, and laboratory data were collected, and patients were followed for 1 year after treatment initiation. Survivors were categorized into the favorable prognosis group (n = 86), and deceased patients into the poor prognosis group (n = 36). Univariate analysis identified age, smoking history, Eastern Cooperative Oncology Group (ECOG) performance status, line of therapy, serum albumin, neutrophil-to-lymphocyte ratio, C-reactive protein (CRP), and carcinoembryonic antigen (CEA) as significant prognostic factors. Multivariate logistic regression revealed that age >60 years, smoking history, ECOG score 2 to 3, hypoalbuminemia (<35 g/L), and elevated CEA (≥5 ng/mL) were independent predictors of poor prognosis. The predictive model demonstrated good discrimination, with an area under the receiver operating characteristic curve (area under the curve) of 0.783 (95% CI: 0.702–0.864) and a concordance index (C-index) of 0.776. Calibration was satisfactory according to the Hosmer–Lemeshow test (*P* = .421). Clinical and laboratory parameters – including age, smoking status, ECOG performance status, albumin, and CEA – provide valuable prognostic information in patients with advanced NSCLC receiving immunotherapy. These factors may assist in risk stratification and individualized treatment planning.

## 1. Introduction

Non-small cell lung cancer (NSCLC) accounts for approximately 85% of all lung cancer cases and remains the leading cause of cancer-related mortality worldwide. Despite significant advances in screening, diagnostic modalities, and systemic therapies, most patients present at advanced stages, when curative surgical resection is no longer feasible. Historically, treatment for advanced NSCLC relied on platinum-based chemotherapy and, more recently, targeted therapies directed at driver mutations such as EGFR, ALK, or ROS1. However, these therapeutic approaches are limited to subsets of patients with specific genetic alterations, leaving a substantial proportion of individuals with restricted treatment options and poor long-term survival outcomes.^[1–3]^ In recent years, the advent of immunotherapy, particularly immune checkpoint inhibitors (ICIs) targeting programmed death-1 (PD-1), programmed death-ligand 1 (PD-L1), and cytotoxic T-lymphocyte–associated protein 4 (CTLA-4), has transformed the treatment landscape of advanced NSCLC. Clinical trials have demonstrated durable responses and survival benefits in subsets of patients, leading to the incorporation of immunotherapy as a standard first-line or subsequent-line treatment. Nonetheless, the clinical benefits of immunotherapy remain heterogeneous, with only a fraction of patients achieving long-term disease control. Primary and acquired resistance to ICIs further complicates treatment efficacy, underscoring the need to identify robust predictors of prognosis and therapeutic response.^[4,5]^

Currently, PD-L1 expression assessed by immunohistochemistry is widely used as a predictive biomarker for ICI efficacy. However, its predictive accuracy is limited by intratumoral heterogeneity, temporal variability, and technical differences in testing platforms. Tumor mutational burden (TMB) and microsatellite instability (MSI) have also been investigated, yet their clinical utility remains restricted due to methodological challenges and inconsistent validation across diverse cohorts. Consequently, reliance on a single biomarker is insufficient, and a more comprehensive evaluation of clinical, pathological, and systemic factors is required to better predict prognosis in patients undergoing immunotherapy.^[6,7]^ Emerging evidence suggests that clinical characteristics, such as performance status (PS), smoking history, metastatic disease burden, and the presence of comorbidities, may significantly influence immunotherapy outcomes. Additionally, systemic inflammatory and nutritional indices, including the neutrophil-to-lymphocyte ratio (NLR), platelet-to-lymphocyte ratio (PLR), and prognostic nutritional index (PNI), have been proposed as cost-effective and readily accessible prognostic markers. These parameters may reflect the host immune status, systemic inflammation, and tumor–host interactions, thereby serving as complementary predictors to molecular biomarkers.^[8,9]^

The present study aims to investigate clinical predictors of prognosis in patients with advanced NSCLC receiving immunotherapy. By systematically evaluating demographic characteristics, baseline clinical parameters, systemic inflammatory markers, and treatment-related factors, this study seeks to delineate variables associated with survival outcomes. A better understanding of these predictors may contribute to refining patient selection criteria, improving therapeutic decision-making, and ultimately enhancing the long-term outcomes of patients with advanced NSCLC treated with immunotherapy.

## 2. Methods

### 2.1. Study design

This study was approved by the Ethics Committee of Xingtai People’s Hospital. This retrospective study evaluated patients diagnosed with advanced NSCLC who received immunotherapy at our institution between August 2020 and August 2024. Eligible patients were required to be 18 years or older at the time of diagnosis, have a histologically or cytologically confirmed diagnosis of advanced or metastatic NSCLC (stage IIIB, IIIC, or IV according to the American Joint Committee on Cancer [AJCC], 8th edition), and have received at least 1 cycle of ICI-based therapy, either as monotherapy or in combination with chemotherapy or targeted agents. Only patients with complete baseline clinical information, including demographic characteristics, smoking history, PS, and laboratory parameters, as well as follow-up data on treatment response, progression-free survival, or overall survival (OS), were included. Patients were excluded if they had a histological diagnosis of small cell lung cancer or mixed small cell/NSCLC histology, had received prior ICI therapy before the current line of treatment, had incomplete clinical records or missing baseline laboratory/imaging data essential for analysis, or had severe autoimmune disease or ongoing systemic immunosuppressive therapy that could interfere with the efficacy assessment of immunotherapy. All patients were followed for 1 year after treatment initiation to record survival outcomes. Survivors at the end of follow-up were categorized into the favorable prognosis group (n = 86), while deceased patients were categorized into the poor prognosis group (n = 36). This study was conducted in accordance with the ethical principles outlined in the Declaration of Helsinki, approved by the institutional medical ethics committee, and written informed consent was obtained from all participants.

### 2.2. Treatment protocol

All enrolled patients received ICI-based therapy in accordance with current clinical practice guidelines and the treating physician’s discretion. ICIs included programmed death-1 (PD-1) inhibitors (e.g., pembrolizumab, nivolumab, tislelizumab, camrelizumab) or programmed death-ligand 1 (PD-L1) inhibitors (e.g., atezolizumab, durvalumab), administered either as monotherapy or in combination with platinum-based doublet chemotherapy or targeted therapy, depending on tumor histology, molecular profile, and baseline clinical status. For patients receiving monotherapy, ICIs were administered intravenously at standard recommended doses and intervals (every 2 or 3 weeks, depending on the specific agent), until documented disease progression, unacceptable toxicity, or withdrawal of consent. For patients receiving combination therapy, ICIs were administered concurrently with platinum-based chemotherapy (cisplatin or carboplatin combined with pemetrexed for nonsquamous histology, or gemcitabine/docetaxel for squamous histology), followed by maintenance therapy as appropriate. Patients harboring driver mutations not amenable to targeted therapy or who had progressed after targeted agents were considered for combination regimens including immunotherapy. Dose modifications, treatment delays, or discontinuations were implemented in cases of grade ≥3 treatment-related adverse events, in accordance with the CTCAE (version 5.0) and standard clinical protocols. Immune-related adverse events (irAEs) were managed with corticosteroids or immunosuppressive agents when indicated. Best supportive care, including antiemetics, analgesics, and nutritional support, was provided as clinically necessary.

### 2.3. Data collection

Clinical and laboratory data were retrospectively collected from electronic medical records. Baseline demographic variables included age (≤60 years vs >60 years) and sex (male vs female). Lifestyle factors comprised smoking history (yes vs no) and alcohol consumption history (yes vs no). Comorbidities were recorded, including hypertension (yes vs no) and diabetes mellitus (yes vs no). Tumor-related characteristics included pathological subtype (adenocarcinoma vs squamous cell carcinoma), clinical stage according to the AJCC (8th edition), and Eastern Cooperative Oncology Group (ECOG) PS (0–1 vs 2–3). Treatment-related parameters included line of therapy (first- or second-line vs third-line and beyond), regimen type (ICI monotherapy vs combination therapy), and duration of immunotherapy. Laboratory indicators collected at baseline comprised serum albumin levels (≥35 g/L vs <35 g/L), hemoglobin levels, NLR, PLR, and PNI. In addition, serum lactate dehydrogenase (LDH), C-reactive protein (CRP), and carcinoembryonic antigen (CEA) levels were obtained, given their potential prognostic value in advanced NSCLC.

### 2.4. Statistical analysis

All statistical analyses were conducted using SPSS software (version 28.0; IBM Corp., Armonk). Continuous variables were expressed as mean ± standard deviation or median with interquartile range (IQR), depending on distribution as assessed by the Kolmogorov–Smirnov test. Categorical variables were summarized as frequencies and percentages. Missing data were assessed before analysis. Variables with incomplete records were handled using complete-case analysis, and no imputation was performed, as the proportion of missing data was low and did not significantly affect the overall dataset. Group comparisons were performed using the Chi-square test or Fisher exact test for categorical variables and the Student *t* test or Mann–Whitney *U* test for continuous variables, as appropriate. Univariate analysis was initially conducted to explore associations between clinical variables and prognosis. Variables with a *P* value <.05 in the univariate analysis were subsequently entered into a multivariate logistic regression model to identify independent predictors. Logistic regression results were presented as odds ratios with 95% confidence intervals. Model discrimination was evaluated using the receiver operating characteristicreceiver operating characteristic (ROC) curve and the area under the curve (AUC), along with the concordance index (C-index). Calibration was assessed using the Hosmer–Lemeshow goodness-of-fit test, with a *P* value >.05 indicating acceptable alignment between predicted and observed outcomes. A 2-sided *P* value <.05 was considered statistically significant.

## 3. Results

### 3.1. Baseline characteristics and follow-up

A total of 122 patients with advanced NSCLC who received immunotherapy were included in the final analysis, comprising 36 patients in the poor prognosis group and 86 patients in the favorable prognosis group. The median age of the study population was 62 years (range, 38–79 years), with 74 males (60.7%) and 48 females (39.3%). A smoking history was present in 49.2% of patients, while 63.1% had a history of alcohol consumption. The most common comorbidities were hypertension (45.1%) and diabetes mellitus (50.8%), whereas chronic obstructive pulmonary disease (22.9%) and cardiovascular disease (26.2%) were less frequent. Adenocarcinoma accounted for 60.7% of cases, and squamous cell carcinoma for 39.3%. At baseline, 75 patients (61.5%) presented with multiple metastatic sites, with brain (25.4%), liver (22.1%), and bone (32.8%) being the most frequent sites of dissemination. ECOG PS scores of 0 to 1 were observed in 63.9% of patients, while 36.1% had ECOG scores of 2 to 3. First- or second-line immunotherapy was administered to 65.6% of patients, whereas 34.4% received third-line treatment or beyond. ICIs were administered either as monotherapy or in combination with chemotherapy, according to clinical indications. The median follow-up duration was 12.4 months (interquartile range [IQR], 10.1–15.6 months). During follow-up, 36 patients (29.5%) died, while 86 patients (70.5%) remained alive at the end of the observation period.

### 3.2. Univariate analysis

Univariate analysis of prognostic factors in patients with advanced NSCLC receiving immunotherapy is summarized in Table 1. Age, smoking history, ECOG PS, line of therapy, serum albumin level, NLR, CRP, and CEA were significantly associated with prognosis. Specifically, patients older than 60 years were more likely to be classified into the poor prognosis group compared with those aged ≤60 years (*P* <.001). A history of smoking was also significantly correlated with unfavorable outcomes (*P* <.001). Higher ECOG scores (2–3) were strongly associated with poor prognosis compared with ECOG scores of 0 to 1 (*P* = .001). In addition, patients who received third-line or later immunotherapy had worse outcomes than those treated in earlier lines (*P* = .009). Regarding laboratory parameters, hypoalbuminemia (<35 g/L) was significantly related to poor prognosis (*P* = .001). Elevated NLR (≥3.0) was also correlated with adverse outcomes (*P* = .013). Similarly, increased CRP levels (≥10 mg/L) were associated with poor prognosis (*P* = .015). Furthermore, elevated CEA levels (≥5 ng/mL) showed a significant association with unfavorable prognosis (*P* = .003). By contrast, sex, alcohol consumption, hypertension, diabetes, COPD, cardiovascular disease, pathological subtype, number of metastatic sites, occurrence of irAEs, hemoglobin, PLR, LMR, LDH, D-dimer, and ALP did not demonstrate statistically significant associations with prognosis (*P* >.05) (Table 1).

**Table 1:** Univariate analysis of prognostic factors in patients with advanced NSCLC receiving immunotherapy.

Variable	Total (n = 122)	Poor prognosis (n = 36)	Favorable prognosis (n = 86)	χ²	*P*
Age					
>60 yr	68	26 (72.2)	42 (36.8)	14.921	<.001
≤60 yr	54	10 (27.8)	44 (63.2)		
Sex					
Male	74	21 (58.3)	53 (49.1)	0.842	.359
Female	48	15 (41.7)	33 (50.9)		
Smoking history					
Yes	60	24 (66.7)	36 (31.6)	13.872	<.001
No	62	12 (33.3)	50 (68.4)		
Alcohol history					
Yes	77	19 (52.8)	58 (52.7)	0.011	.918
No	45	17 (47.2)	28 (47.3)		
Hypertension					
Yes	55	16 (44.4)	39 (36.0)	0.472	.492
No	67	20 (55.6)	47 (64.0)		
Diabetes					
Yes	62	14 (38.9)	48 (41.9)	0.152	.696
No	60	22 (61.1)	38 (58.1)		
COPD					
Yes	28	9 (25.0)	19 (22.1)	0.311	.577
No	94	27 (75.0)	67 (77.9)		
Cardiovascular disease					
Yes	32	10 (27.8)	22 (25.6)	0.402	.526
No	90	26 (72.2)	64 (74.4)		
Pathology					
Adenocarcinoma	74	18 (50.0)	56 (51.8)	0.027	.870
Squamous carcinoma	48	18 (50.0)	30 (48.2)		
Metastatic sites					
Single site	47	15 (41.7)	32 (37.2)	2.114	.146
Multiple sites	75	21 (58.3)	54 (62.8)		
ECOG score					
0–1	78	11 (30.6)	67 (62.8)	11.287	.001
2–3	44	25 (69.4)	19 (37.2)		
Line of therapy					
First-/second-line	80	13 (36.1)	67 (62.1)	6.771	.009
Third-line or beyond	42	23 (63.9)	19 (37.9)		
irAEs					
Yes	41	10 (27.8)	31 (36.0)	2.108	.147
No	81	26 (72.2)	55 (64.0)		
Albumin (g/L)					
≥35	79	10 (27.8)	69 (60.7)	10.983	.001
<35	43	26 (72.2)	17 (39.3)		
Hemoglobin					
Normal (≥120 g/L)	81	23 (63.9)	58 (67.4)	0.712	.399
Low (<120 g/L)	41	13 (36.1)	28 (32.6)		
NLR					
High (≥3.0)	56	24 (66.7)	32 (37.2)	6.214	.013
Low (<3.0)	66	12 (33.3)	54 (62.8)		
PLR					
High (≥150)	64	21 (58.3)	43 (50.0)	1.992	.158
Low (<150)	58	15 (41.7)	43 (50.0)		
LMR					
Low (<2.5)	52	18 (50.0)	34 (39.5)	0.814	.367
High (≥2.5)	70	18 (50.0)	52 (60.5)		
LDH (U/L)					
Elevated (≥250)	49	17 (47.2)	32 (37.2)	1.286	.257
Normal (<250)	73	19 (52.8)	54 (62.8)		
CRP (mg/L)					
Elevated (≥10)	54	22 (61.1)	32 (37.2)	5.917	.015
Normal (<10)	68	14 (38.9)	54 (62.8)		
D-dimer (mg/L)					
Elevated (≥0.5)	57	20 (55.6)	37 (43.0)	0.892	.345
Normal (<0.5)	65	16 (44.4)	49 (57.0)		
ALP (U/L)					
Elevated (≥120)	46	15 (41.7)	31 (36.0)	0.912	.339
Normal (<120)	76	21 (58.3)	55 (64.0)		
CEA (ng/mL)					
Elevated (≥5)	66	26 (72.2)	40 (46.5)	8.621	.003
Normal (<5)	56	10 (27.8)	46 (53.5)		

ALP = alkaline phosphatase, CEA = carcinoembryonic antigen, CRP = C-reactive protein, ECOG = Eastern Cooperative Oncology Group, irAEs = immune-related adverse events, LDH = lactate dehydrogenase, LMR = lymphocyte-to-monocyte ratio, NLR = neutrophil-to-lymphocyte ratio, NSCLC = non-small cell lung cancer, PLR = platelet-to-lymphocyte ratio.

### 3.3. Multivariate logistic regression analysis

Multivariate logistic regression analysis was performed to identify independent prognostic predictors among variables with significance in the univariate analysis. As shown in Table 2, age >60 years (OR = 2.409, 95% CI: 1.140–5.092, *P* = .020), smoking history (OR = 2.480, 95% CI: 1.126–5.462, *P* = .022), ECOG PS of 2 to 3 (OR = 3.523, 95% CI: 1.551–7.993, *P* = .003), low serum albumin level (<35 g/L) (OR = 3.025, 95% CI: 1.313–7.352, *P* = .010), and elevated CEA level (≥5 ng/mL) (OR = 2.583, 95% CI: 1.165–5.726, *P* = .021) were identified as independent risk factors for poor prognosis. In contrast, line of therapy, NLR, and CRP did not retain statistical significance after adjustment (*P* >.05). These findings suggest that older age, smoking history, impaired PS, hypoalbuminemia, and elevated tumor markers are robust predictors of unfavorable outcomes in patients with advanced NSCLC treated with immunotherapy (Table 2).

**Table 2:** Multivariate logistic regression analysis of prognostic factors in patients with advanced NSCLC receiving immunotherapy.

Factors	β value	SE value	Wald value	OR value	95% CI for OR	*P*-value
Age (>60 vs ≤60)	0.879	0.376	5.464	2.409	1.140–5.092	.020
Smoking history (Yes vs No)	0.908	0.398	5.205	2.480	1.126–5.462	.022
ECOG score (2–3 vs 0–1)	1.259	0.419	9.017	3.523	1.551–7.993	.003
Line of therapy (≥3 vs ≤2)	0.518	0.406	1.628	1.679	0.737–3.825	.202
Albumin (<35 g/L vs ≥35 g/L)	1.107	0.428	6.691	3.025	1.313–7.352	.010
NLR (≥3 vs <3)	0.459	0.392	1.373	1.583	0.730–3.432	.241
CRP (≥10 vs <10)	0.409	0.379	1.162	1.505	0.710–3.188	.281
CEA (≥5 vs <5)	0.949	0.410	5.360	2.583	1.165–5.726	.021

CEA = carcinoembryonic antigen, CI = confidence interval, CRP = C-reactive protein, ECOG = Eastern Cooperative Oncology Group, NLR = neutrophil-to-lymphocyte ratio, NSCLC = non-small cell lung cancer, OR = odds ratio, SE = standard error.

### 3.4. Model discrimination and calibration

The multivariate logistic regression model demonstrated acceptable discriminatory ability for predicting prognosis in patients with advanced NSCLC treated with immunotherapy. The AUC was 0.783 (95% CI: 0.702–0.864), indicating good accuracy in distinguishing between patients with favorable and poor prognosis. The C-index of the model was 0.776, further supporting its predictive performance. Calibration of the model was evaluated using the Hosmer–Lemeshow goodness-of-fit test, which showed no significant deviation between predicted and observed outcomes (*P* = .421), suggesting satisfactory model fit.

## 4. Discussion

This study identified a parsimonious set of readily obtainable clinical variables – age, smoking history, ECOG PS, serum albumin, and CEA – as independent predictors of prognosis in patients with advanced NSCLC treated with ICIs. Our findings underscore the persistent and crucial role of host-related constitutional and systemic factors in shaping outcomes within the immunotherapy era. The multivariable model incorporating these 5 routine parameters demonstrated acceptable discrimination (AUC 0.783; C-index 0.776) and satisfactory calibration, supporting its feasibility for pragmatic risk stratification in diverse clinical settings.

The primary novelty and clinical relevance of our work lie in developing and internally validating a simple yet effective prognostic tool using universally available, low-cost variables. This model holds significant translational potential, particularly for rapid risk assessment and individualized treatment planning in resource-limited settings or situations where sophisticated molecular profiling is unavailable, inconclusive, or its results are pending.

Older age (>60 years) was independently associated with a higher risk of poor prognosis. Aging is accompanied by well-documented immunological decline, including diminished naïve T-cell reserves, a skewed T-cell receptor repertoire, and a state of low-grade chronic inflammation (“inflammaging”), all of may attenuate antitumor immunity and potentially limit the effectiveness of PD-(L)1 blockade. While selected older adults can undoubtedly derive significant benefit from ICIs, our data suggest that chronological age, serving as a surrogate for immune senescence and underlying frailty, retains independent prognostic value even after adjusting for other key clinical factors.^[10,11]^ This finding argues strongly for incorporating careful geriatric and functional assessments into the decision-making process when selecting candidates for immunotherapy.

A history of smoking emerged as an independent negative prognostic factor. Biologically, chronic tobacco exposure induces persistent airway and systemic inflammation, endothelial dysfunction, and broad immune dysregulation. These detrimental host effects may counterbalance any potential immunogenic advantage conferred by the higher TMB typically observed in smokers.^[10,11]^ Our observation that smoking history remained significant after multivariate adjustment highlights that long-standing, tobacco-induced host alterations can shape ICI responses independently of tumor genomics. This supports the integration of smoking status into prognostic models and underscores the critical importance of implementing robust tobacco cessation strategies concurrent with anticancer therapy.

ECOG performance status (PS) of 2 to 3 was one of the strongest predictors, conferring a more than threefold increased risk of poor prognosis compared to PS 0 to 1. A poor PS likely reflects a cumulative impairment of physiological reserve, compromised immunity, a higher burden of comorbidities, and reduced tolerance to treatment intensity – all of which can diminish the capacity to achieve durable disease control with ICIs.^[12]^ This finding powerfully reinforces the established, yet sometimes underemphasized, principle that PS must remain a cornerstone of therapeutic decision-making and intensity tailoring in the ICI landscape.

Among laboratory parameters, hypoalbuminemia (<35 g/L) remained independently associated with poor outcomes. Serum albumin serves as an integrative marker of nutritional status, systemic inflammatory response, and hepatic synthetic function; low levels are frequently intertwined with sarcopenia, impaired cellular immunity, and heightened vulnerability to treatment-related toxicity.^[13–15]^ Similarly, an elevated CEA level (≥5 ng/mL) independently predicted adverse prognosis, consistent with its established role as a surrogate for tumor burden and aggressiveness, and potentially as an indicator of an immune-evasive tumor microenvironment.^[16,17]^ In contrast, NLR and CRP lost statistical significance in the multivariate model, suggesting that their prognostic information is largely captured by the more comprehensive measures of PS and albumin. This observation argues for prioritizing composite clinical-biochemical profiles over isolated inflammatory ratios when constructing practical prognostic tools for routine care.

The model’s performance characteristics underscore its potential clinical utility. The AUC and C-index indicate that this compact 5-variable panel can stratify patients into higher versus lower risk categories with reasonable accuracy. The nonsignificant Hosmer–Lemeshow test suggests good calibration across risk strata. While external validation is mandatory, these performance metrics are comparable to other pragmatic prognostic algorithms in immuno-oncology and hint at potential for bedside application in patient counseling and personalized treatment planning.

Our results align with contemporary evidence reaffirming the prognostic value of host factors, nutritional status, and conventional tumor markers in NSCLC immunotherapy.^[13–17]^ The distinctive clinical implication of our model is its demonstration that a minimal set of easily accessible variables can provide substantial prognostic information, offering a practical and immediately implementable stratification tool.

The identification of these 5 independent predictors enables a pragmatic risk stratification approach at treatment initiation. Patients with unfavorable profiles (e.g., elderly smokers with poor PS, hypoalbuminemia, and high CEA) may be candidates for intensified supportive care (including nutritional optimization and prehabilitation), closer monitoring, early palliative care involvement, or consideration of combination regimens or clinical trial enrollment. Conversely, patients with favorable profiles might proceed with ICI-based strategies with a greater expectation of benefit and potentially fewer adjunctive interventions. Incorporating these variables into standardized clinical assessment can improve prognostic discussions, better align treatment intensity with individual physiologic reserve, and optimize resource allocation, particularly in settings with limited access to advanced biomarker testing. Such steps could ultimately enhance the real-world effectiveness and cost-efficiency of immunotherapy programs.

This study has several strengths, including its focus on universally available variables, enhancing generalizability and immediate translational potential; a robust analytical approach from univariate screening to multivariate modeling to limit confounding; and a comprehensive reporting of model performance using both discrimination and calibration metrics.

Nevertheless, these findings must be interpreted considering several limitations. The retrospective, single-center design inherently introduces potential selection bias and limits the generalizability of the findings. The relatively modest sample size and potential for unmeasured confounding (e.g., detailed comorbidity indices, specific ICI agents) may affect the reliability of the associations. The 1-year follow-up period may underestimate long-term survival and could misclassify patients with delayed benefits from ICIs. This timeframe also precluded the use of more robust time-to-event analyses (e.g., Cox regression). The lack of external validation curtails the robustness and broad clinical applicability of our predictive model. Furthermore, the absence of key tumor-intrinsic biomarkers – most notably PD-L1 expression, TMB, and driver mutation status – means their potential confounding or complementary predictive role could not be assessed. Future research should be prospective, multi-center, involve larger cohorts with longer follow-up, include external validation, and integrate molecular, imaging, and serial biomarker data to further refine, validate, and potentially enhance this clinically accessible prognostic model.

## 5. Conclusions

This study demonstrated that older age, smoking history, impaired PS, low serum albumin, and elevated CEA are independent predictors of unfavorable prognosis in advanced NSCLC patients treated with immunotherapy. The predictive model showed reliable discrimination and calibration, indicating potential value for clinical risk stratification. These results emphasize the importance of incorporating clinical and laboratory parameters into individualized treatment planning.

## Author contributions

**Conceptualization:** Zhian Qiao, Lin Pang, Hongxin Zheng.

**Data curation:** Zhian Qiao, Lin Pang, Hongxin Zheng.

**Formal analysis:** Zhian Qiao, Lin Pang, Hongxin Zheng.

**Funding acquisition:** Zhian Qiao, Lin Pang.

**Investigation:** Zhian Qiao, Hongxin Zheng.

**Writing – original draft:** Zhian Qiao, Lin Pang, Hongxin Zheng.

**Writing – review & editing:** Zhian Qiao, Lin Pang, Hongxin Zheng.
